# The Impact of Carbohydrate Management on Coleoptile Elongation in Anaerobically Germinating Seeds of Rice (*Oryza sativa* L.) under Light and Dark Cycles

**DOI:** 10.3390/plants12071565

**Published:** 2023-04-05

**Authors:** Haru Hirano, Takeru Watanabe, Mika Fukuda, Takeshi Fukao

**Affiliations:** Department of Bioscience and Biotechnology, Fukui Prefectural University, Fukui 910-1195, Japan

**Keywords:** anaerobic germination, coleoptile elongation, *Oryza sativa*, oxygen deprivation, submergence, sugar sensitivity

## Abstract

The ability of rice to elongate coleoptiles under oxygen deprivation is a determinant of anaerobic germination tolerance, critical for successful direct seeding. Most studies on anaerobic coleoptile elongation have been performed under constant darkness or in flooded soils because a drilling method was the primary approach for direct seeding of rice. However, aerial seeding is becoming popular, in which seeds which land on flooded soils are exposed to light during the daytime. Here, we investigated physiological mechanisms underlying anaerobic elongation of coleoptiles under light and dark cycles. This study identified two novel varieties, LG and L202, enabling the development of long coleoptiles under oxygen limitation, comparable to well-characterized varieties with strong anaerobic germination tolerance. Germination experiments using these two tolerant and two intolerant varieties, including Takanari and IR64, revealed that light and dark cycles increased coleoptile length in LG, Takanari, and IR64 relative to constant darkness. Interestingly, even in intolerant lines, dramatic starch breakdown and soluble carbohydrate accumulation occurred under oxygen limitation. However, intolerant lines were more susceptible to a representative soluble sugar, glucose, than tolerant lines under oxygen deprivation, suggesting that coleoptile growth can be inhibited in intolerant lines due to hypersensitivity to soluble sugars accumulated in anaerobically germinating seeds.

## 1. Introduction

Direct seeding is an effective labor-saving approach for rice production, allowing farmers to omit seedling culture and transplanting, the most labor-intensive steps. However, direct seeding is not widespread in most rice-producing countries due to the lack of anaerobic germination tolerance in their commonly grown cultivars. Anaerobic growth of coleoptiles is a determinant for this tolerance because only coleoptiles can emerge and elongate under submergence until the tip of this organ reaches near the water surface containing more oxygen [1].

Mechanisms regulating anaerobic elongation of coleoptiles in rice have been investigated for several decades. It has been recognized that coleoptiles of rice seedlings germinated under oxygen deprivation are stark white even in the light due to the absence of protochlorophyllide and chlorophylls [2,3]. Thus, anaerobic growth of coleoptiles does not rely on photosynthesis, and carbohydrate reserves in endosperms are the solo energy sources supporting rapid coleoptile elongation. Unlike other cereals, rice can break down starch into soluble carbohydrates in endosperms without oxygen, enabling this wetland species to germinate under anoxia [4,5]. It was also shown that the degree of starch breakdown under oxygen deprivation positively correlated with the length of coleoptiles in anaerobically germinating seeds of rice [6,7].

Signaling cascades regulating starch catabolism during anaerobic germination have been uncovered in rice. α-amylases are key enzymes degrading starch during anaerobic germination, whose expression is directly regulated by MYELOBLASTOSIS SUCROSE 1 (MYBS1) transcription factor [8,9]. Physical interaction of MYBS1 with the α-amylase promoter is activated through phosphorylation of MYBS1 by SUCROSE NONFERMENTING 1-RELATED PROTEIN KINASE 1A (SnRK1A) [10,11]. Protein accumulation of SnRK1A is upregulated by CALCINEURIN B-LIKE PROTEIN-INTERACTING Ser/Thr PROTEIN KINASE 15 (CIPK15), which is induced under sugar-starved and oxygen-depleted conditions [11]. The nuclear localization of SnRK1A and subsequent starch degradation mediated by MYBS1 are inhibited by hypoxia-inducible SnRK1A-INTERACTING NEGATIVE REGULATOR 1 and 2 (SKIN1/2) [12]. MYBS1-mediated expression of α-amylases is also suppressed by MYBS2, which competes with MYBS1 for promoter binding [13].

Genes and loci affecting anaerobic germination tolerance have also been identified by quantitative trait locus (QTL) analysis in rice. Five QTLs positively affecting anaerobic germination tolerance were detected in a representative tolerant variety, Khao Hlan On [14]. Of these QTLs, *qAG-9-2* (*AG1*) with the most significant LOD score and phenotypic variance encoded TREHALOSE-6-PHOSPHATE PHOSPHATASE 7 (TPP7), suppressing trehalose-6-phosphate (T6P)-mediated inhibition of SnRK1A activity and starch degradation in anaerobically germinating seeds [15]. Although the tolerant allele of *TPP7* was identified in a *japonica* variety, Khao Hlan On, a large-scale survey of temperate and tropical *japonica* accessions revealed that polymorphisms and transcriptional variations of *TPP7* are not associated with coleoptile elongation under oxygen deprivation, suggesting that anaerobic germination tolerance can also be regulated by *TPP7*-independent mechanisms [16]. Another strong QTL contributing to anaerobic germination tolerance, *qAG7.1* (*AG2*), was found in a highly tolerant variety Ma-Zhan Red [17]. *AG1* (*TPP7*) and *AG2* were introgressed into multiple backgrounds, resulting in enhanced starch degradation, soluble sugar accumulation, and anaerobic germination tolerance with no adverse effects on seed physiology [7,18]. Recently, a combination of genome-wide association studies (GWAS) and post-GWAS analysis identified several genes involved in anaerobic germination tolerance, one of which was *CLASSY1* (*CLSY1*), a gene involved in the RNA-directed DNA methylation pathway [19]. A mutation in *CLSY1* altered methylation profiles and subsequent gene expression patterns, leading to enhanced anaerobic germination and seedling establishment under submergence.

These studies on anaerobic germination and coleoptile elongation have been performed under constant darkness or flooded field conditions where seeds are buried in the soil. This is because a drilling method in which light is not available during germination was the primary approach for direct seeding of rice. However, aerial seeding, in which seeds are broadcasted on flooded soils by aerial vehicles, is becoming popular due to better weed control and lower exposure to animal pests [20,21]. In this method, seeds landed on the soil surface are exposed to light during the daytime. Here, we evaluated the impact of carbohydrate management and relevant hormones on anaerobic coleoptile elongation in four rice varieties with contrasting tolerance to anaerobic germination under light and dark cycles. This study provides new insight into the regulatory mechanisms underlying anaerobic germination tolerance in rice when light is available.

## 2. Results

### 2.1. Screening of Rice Varieties Regarding Coleoptile Elongation under Oxygen Deprivation

**Figure 1:**
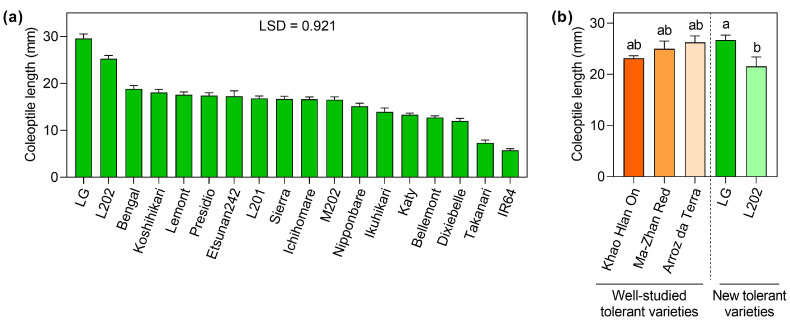
The distinct capability of rice accessions for coleoptile elongation under oxygen deficiency. (**a**) The coleoptile length of 18 rice varieties germinated under anaerobic conditions for 5 days under 12 h light and 12 h dark cycles. Data represent means ± SE (*n* = 15). Multiple comparisons of means were performed using ANOVA followed by Fisher’s least significant difference (LSD) test. (**b**) Coleoptile elongation under oxygen deprivation in well-studied tolerant varieties vs. tolerant ones characterized in this study. Growth conditions used in (**b**) were identical to those in (**a**). Data represent means ± SE (*n* = 20). Multiple comparisons of means were performed using ANOVA followed by Tukey’s honest significant test. Bars not sharing the same letter are significantly different (*p* < 0.05).

The degree of coleoptile elongation under low oxygen has been used as an indicator for anaerobic germination tolerance in rice [11,15]. To identify new tolerant varieties which can serve as novel genetic resources for anaerobic germination studies, we compared 18 rice accessions in terms of coleoptile elongation under oxygen deprivation (Figure 1a). Seeds were incubated in sealed glass vials filled with deoxygenated water under 12 h light and 12 h dark cycles for 5 days. Of these varieties, LG formed the longest coleoptiles, whereas IR64 had the shortest ones. The difference in coleoptile length between the two genotypes was approximately five-fold. Restricted elongation of coleoptiles in IR64 and other genotypes with short coleoptiles may result from seed dormancy and slow shoot elongation that can be observed even under non-stress (aerobic) conditions. To evaluate these possibilities, the percent germination and shoot elongation of 18 rice accessions were assessed under aerobic conditions. No genotypes appeared to be dormant (Figure S1). In addition, varieties with the shortest coleoptiles under anaerobic conditions, such as IR64 and Takanari, formed longer shoots than most lines under aerobic conditions (Figure S2). These data demonstrate that restricted elongation of coleoptiles under low oxygen in IR64 and Takanari is not attributable to seed dormancy and slow shoot elongation that can be observed under aerobic conditions. Based on these results, we determined to utilize these two varieties as intolerant lines in this study.

Of the 18 accessions surveyed, LG and L202 showed the longest coleoptiles under oxygen deprivation, selected as tolerant lines in this study. To discern whether this capability is comparable to that in well-characterized tolerant lines, we conducted anaerobic germination tests using Khao Hlan On, Ma-Zhan Red, and Arroz da Terra [14,17,22] along with our tolerant lines, LG and L202 (Figure 1b). Consistent with the result in Figure 1a, coleoptile elongation under oxygen deprivation in LG was significantly greater than in L202. The ability of LG and L202 for anoxic coleoptile elongation was equivalent to that in the three well-characterized tolerant lines. The data indicate that LG and L202 identified in this study can serve as new genetic resources for a mechanistic understanding and gene discovery for anaerobic germination tolerance in rice.

### 2.2. Effect of Light on Coleoptile Elongation under Oxygen Deprivation

**Figure 2:**
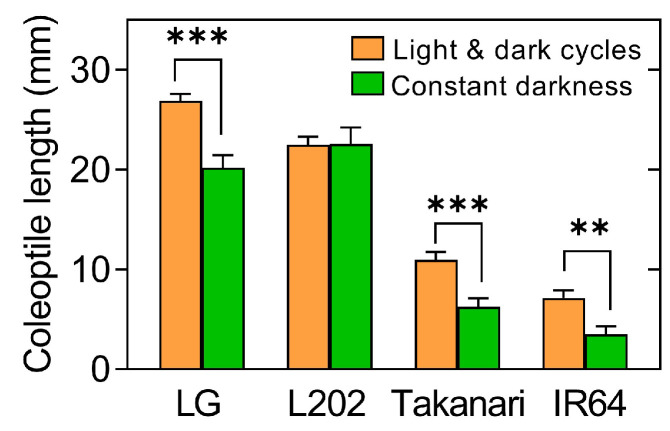
The influence of light on coleoptile elongation under oxygen deprivation in rice varieties, with and without anaerobic germination tolerance. Seeds were germinated under oxygen deprivation for 5 days under 12 h light and 12 h dark cycles or under constant darkness. Data represent means ± SE (*n* = 20). Asterisks indicate a significant difference between the two growth conditions (** *p* < 0.01, *** *p* < 0.001; *t*-test).

Most studies on anaerobic germination tolerance have been carried out under constant darkness or field conditions in which seeds are buried in flooded soil. In this study, germination experiments were performed under 12 h light and 12 h dark cycles because seeds sown by aerial seeding receive light in flooded fields. To evaluate the influence of light on anaerobic elongation of coleoptiles in rice, representative tolerant and intolerant varieties were subjected to anaerobic germination experiments under light-dark cycles and constant darkness (Figure 2). Tolerant lines, LG and L202, had longer coleoptiles than intolerant lines, Takanari and IR64, under both conditions. Interestingly, coleoptile lengths in LG, Takanari, and IR64 were longer under light and dark cycles than under constant darkness. In L202, coleoptile length was unaltered by changing light conditions. This result indicates that light and dark cycles can positively affect coleoptile elongation under limited oxygen, depending on varieties.

### 2.3. Effect of Growth-Promoting Hormones on Coleoptile Elongation under Oxygen Deprivation

**Figure 3:**
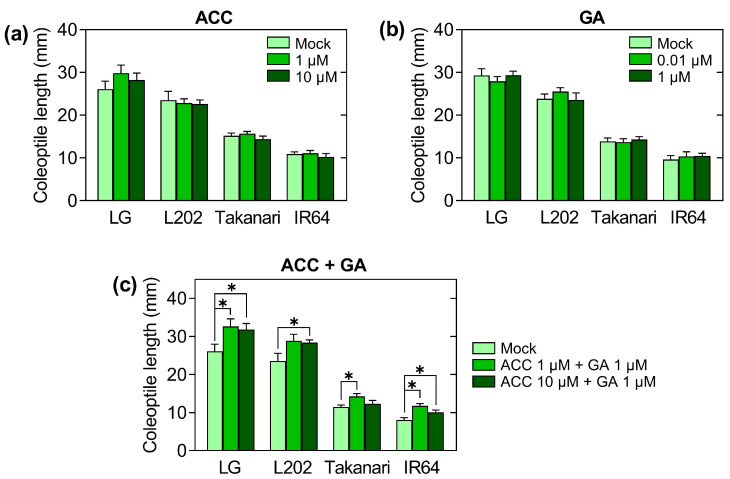
The effect of growth-promoting hormones on coleoptile elongation under oxygen deprivation in rice varieties with and without anaerobic germination tolerance. Seeds were incubated under 12 h light and 12 h dark cycles for 5 days in deoxygenated water containing an ethylene precursor, 1-aminocyclopropane-1-carboxylate (ACC) (**a**), gibberellic acid (GA) (**b**), or a mixture of both (**c**). Data represent means ± SE (*n* = 20). Asterisks indicate a significant difference between mock and hormone-treated samples (* *p* < 0.05; *t*-test).

Phytohormones are critical regulators of growth and adaptive responses to various environmental conditions. For instance, ethylene performs a pivotal role in triggering stress responses to submergence, including leaf elongation at the vegetative stage in rice [23,24]. Additionally, exogenous ethylene application promotes shoot elongation in rice seeds germinating in the dark under aerobic conditions [25]. In our experimental system, the gaseous hormone ethylene cannot be applied to germinating seeds in water. Therefore, the immediate precursor of ethylene, 1-aminocyclopropane-1-carboxylate (ACC), was used instead of ethylene (Figure 3a). ACC treatment did not affect shoot elongation under low oxygen in either tolerant or intolerant genotypes.

Under aerobic conditions, gibberellins (GAs) promote seed germination and subsequent shoot elongation in plants, including rice [26,27]. To discern the role of GA in coleoptile elongation under oxygen deprivation, representative tolerant and intolerant varieties were anaerobically germinated in deoxygenated water containing GA (Figure 2b). Unlike aerobic germination, GA did not affect shoot elongation under low oxygen in tolerant or intolerant genotypes.

Combined application of ACC and GA promoted the elongation of coleoptiles and other shoot tissues in rice under submergence, relative to ACC and GA alone [28]. In this system, mesocotyles, first leaves, second leaves, and coleoptiles elongated, whereas only coleoptiles appeared in our system. These observations imply that the oxygen concentration in their system is higher than in our system (1 ppm of oxygen). However, the combined application of these growth-promoting hormones may also have a synergistic effect under our growth conditions. To test this, we put these hormones in deoxygenated water and conducted anaerobic germination experiments (Figure 3c). This analysis revealed that the combined application of ACC and GA significantly promotes coleoptile elongation at 1 ppm of oxygen, even though the application of ACC and GA alone did not affect it.

### 2.4. Role of Starch Breakdown and Soluble Carbohydrate Accumulation in Coleoptile Elongation under Oxygen Deprivation

**Figure 4:**
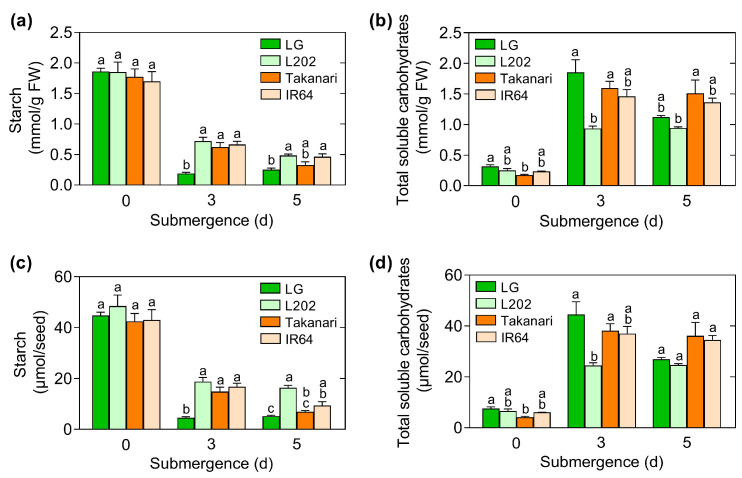
Starch breakdown and soluble carbohydrate accumulation in anaerobically germinating seeds. Seeds were incubated under 12 h light and 12 h dark cycles for up to 5 days under oxygen deprivation. The concentrations of starch (**a**,**c**) and total soluble carbohydrates (**b**,**d**) in seeds or seedlings were quantified on days 0, 3, and 5. In (**a**) and (**c**), the concentrations were calculated on a per g fresh weight (FW) basis. In (**b**) and (**d**), the concentrations were determined on a per seed basis. Data represent means ± SE (*n* = 3). Multiple comparisons of means were performed using ANOVA followed by Tukey’s honest significant test. Bars not sharing the same letter are significantly different at each time point (*p* < 0.05).

Photosynthesis does not occur in anaerobically germinating seeds because protochlorophyllide and chlorophylls are absent, and carbon dioxide is not produced by respiration [2,3]. Thus, seeds and seedlings are heterotrophic under anaerobic conditions, which rely on carbohydrate reserves in endosperms for coleoptile growth. To monitor the levels of starch breakdown and soluble carbohydrate accumulation in anaerobically germinating seeds of tolerant and intolerant lines, carbohydrate assays were carried out (Figure 4). The concentrations of starch are not distinct in tolerant and intolerant accessions on day 0 (Figure 4a). On day 3, the levels of starch dramatically reduced in all genotypes. The starch breakdown in LG was more rapid than any others. On days 3 and 5, starch concentration in tolerant L202 was similar to those in intolerant Takanari and IR64. Consistent with the dramatic degradation of starch, the concentrations of total soluble carbohydrates were markedly elevated in all genotypes (Figure 4b). On days 3 and 5, the levels of total soluble carbohydrates in intolerant Takanari and IR64 were comparable to that in tolerant LG.

We also calculated the amount of starch and total soluble carbohydrates per seed (Figure 4c,d) because these values can be critical traits influencing the degree of coleoptile elongation under oxygen deprivation. Consistent with the fresh weight basis data, there were no significant differences in the amount of starch per seed among the four genotypes on day 0. LG also rapidly catabolized starch on day 3, relative to other accessions. Unlike the fresh weight basis data, the amount of starch per seed in intolerant Takanari was lower than that in tolerant L202 on day 5, suggesting that intensive breakdown of starch occurred in the intolerant line. The amount of total soluble carbohydrates per seed was also compared with the fresh weight basis data. The only difference is that the level of total soluble carbohydrates was greater in Takanari than L202 on day 5 in the fresh weight basis data, whereas these values were not significantly distinct in the per seed basis data. Altogether, these results demonstrate that the dramatic breakdown of starch occurs in anaerobically germinating seeds of both tolerant and intolerant accessions. It is most likely that the level of starch degradation is not closely associated with the degree of anaerobic coleoptile elongation under our growth conditions.

### 2.5. Effect of Exogenous Glucose on Coleoptile Elongation under Oxygen Deprivation

**Figure 5:**
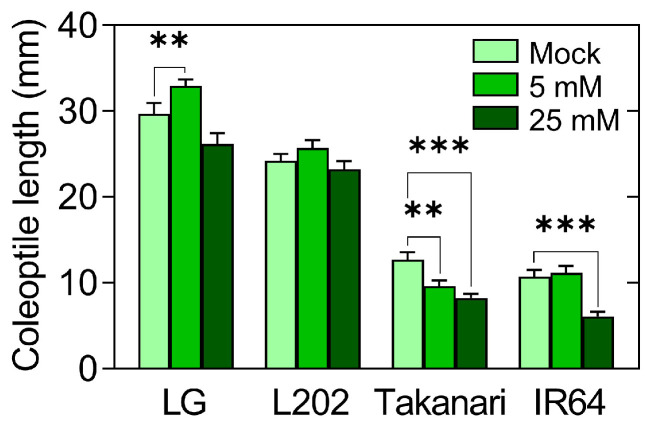
The influence of exogenous glucose on coleoptile elongation under oxygen deprivation in rice varieties with and without anaerobic germination tolerance. Seeds were incubated under 12 h light and 12 h dark cycles for 5 days in deoxygenated water containing 0 mM (mock), 5 mM, and 25 mM glucose. Data represent means ± SE (*n* = 20). Asterisks indicate a significant difference between mock and glucose-treated samples (** *p* < 0.01, *** *p* < 0.001; *t*-test).

The ability of rice to germinate without oxygen depends on the capability of this wetland species to degrade starch into soluble carbohydrates even in the absence of oxygen [29,30]. In fact, upland crops, such as wheat and barley, are unable to germinate under oxygen deprivation due to the failure of starch breakdown [4,5]. Not surprisingly, the exogenous application of glucose to wheat seeds made it possible to germinate under anoxia [31]. Although the substantial breakdown of stored starch occurred in anaerobically germinating seeds of all four accessions (Figure 4a,c), the application of exogenous sugars may differentially affect anaerobic coleoptile elongation among tolerant and intolerant varieties. To evaluate this possibility, anaerobic germination tests were carried out by supplementing deoxygenated water with glucose (Figure 5). Exogenous glucose at 5 mM increased coleoptile elongation in tolerant LG, whereas it reduced coleoptile length in intolerant Takanari. Applying 25 mM glucose did not affect coleoptile growth in tolerant LG, but reduced coleoptile length in intolerant Takanari and IR64. No glucose effect was observed in L202 at either concentration.

### 2.6. Impact of Glucose Sensitivity on Coleoptile Elongation under Oxygen Deprivation

**Figure 6:**
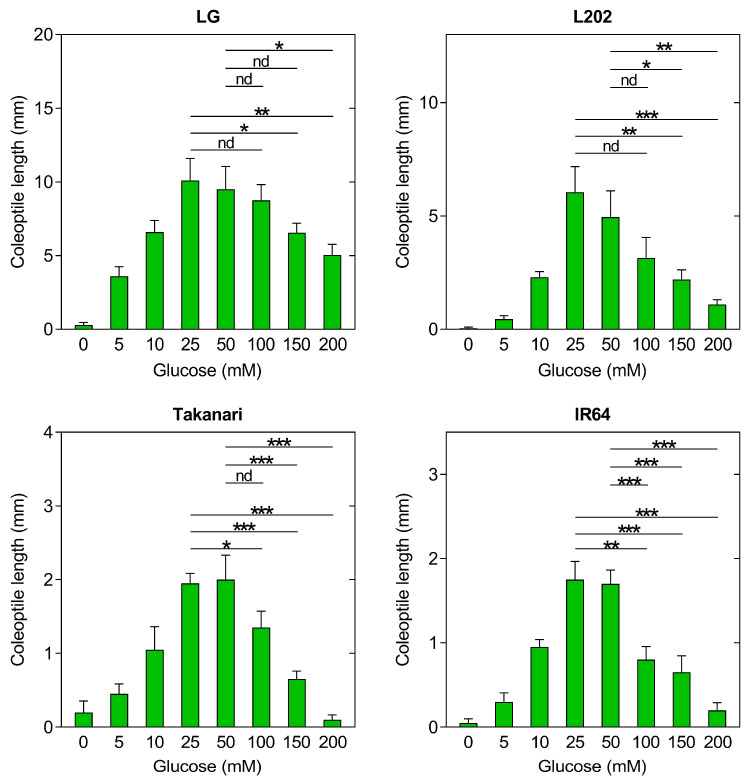
The impact of exogenous glucose on coleoptile elongation under oxygen deprivation in endosperm-less seeds of rice varieties with and without anaerobic germination tolerance. The endosperm of each seed was removed and incubated under 12 h light and 12 h dark cycles for 5 days in deoxygenated water containing 0 mM (mock) to 200 mM glucose. Data represent means ± SE (*n* = 20). Asterisks indicate a significant difference between two data sets indicated by a line (* *p* < 0.05, ** *p* < 0.01, *** *p* < 0.001; *t*-test). nd, no significant difference.

The application of exogenous glucose showed antithetical effects on anaerobic elongation of coleoptiles in tolerant vs. intolerant lines. Excessive accumulation of glucose within a germinating seed can lead to restricted elongation of the coleoptile because even glucose, an energy resource, can inhibit plant growth when the concentrations reach toxic levels [32,33,34]. In anaerobically germinating seeds, soluble carbohydrates were highly accumulated in both tolerant and intolerant lines (Figure 4b,d), and glucose supplementation to these seeds can increase the internal concentrations of soluble sugars to toxic levels. It can be expected that intolerant lines are more sensitive to glucose toxicity than tolerant lines, thereby stunting coleoptile growth in intolerant lines. Based on these observations, we hypothesized that restricted elongation of coleoptiles in intolerant lines could result from hypersensitivity to soluble sugars highly accumulated during anaerobic germination. To test this hypothesis, endosperm-less seeds were subjected to germination tests in deoxygenated water containing a physiological range of glucose concentrations (Figure 6). This analysis used endosperm-less seeds because if intact seeds are used, varied levels of endogenous glucose are accumulated in germinating seeds of the four accessions, which makes it difficult to interpret the impact of glucose on anaerobic coleoptile growth in each accession. In all genotypes, coleoptile growth was considerably repressed when endosperms were removed, confirming that carbohydrates provided by endosperms are vital for anaerobic germination. However, glucose supplementation increased coleoptile growth in a dose-dependent manner until the concentrations reached 25 mM to 50 mM. Notably, increased concentrations of glucose to 100 mM, 150 mM, and/or 200 mM reduced coleoptile elongation, with more severe reductions in anaerobic germination intolerant lines than tolerant lines. For example, in tolerant lines, such as LG and L202, coleoptile lengths at 100 mM glucose were not significantly different from those at 25 mM or 50 mM. In intolerant Takanari and IR64, however, the lengths at 100 mM glucose were significantly lower than those at 25 mM and/or 50 mM glucose, indicating hypersensitivity to glucose in intolerant lines.

**Figure 7:**
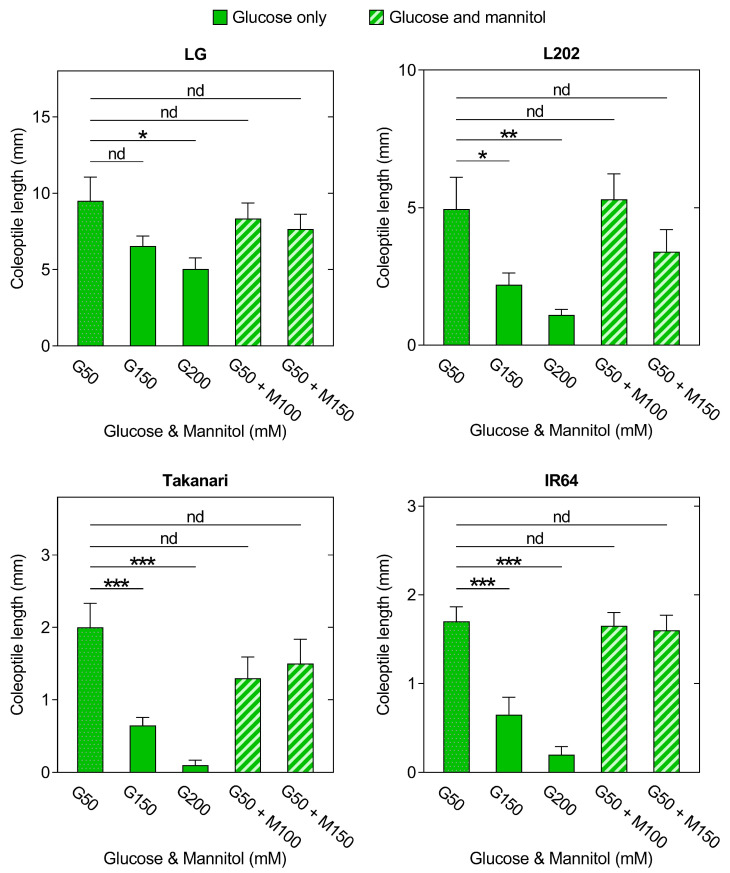
Evaluation of the osmotic effect on glucose-induced suppression of coleoptile elongation under oxygen deprivation in endosperm-less seeds of rice varieties with and without anaerobic germination tolerance. The endosperm of each seed was removed and incubated under 12 h light and 12 h dark cycles for 5 days in deoxygenated water containing glucose or glucose + mannitol. Data represent means ± SE (*n* = 20). Asterisks indicate a significant difference between two data sets indicated by a line (* *p* < 0.05, ** *p* < 0.01, *** *p* < 0.001; *t*-test). nd, no significant difference; G, glucose; M, mannitol.

To discern whether growth inhibition at high glucose concentrations did not result from osmotic stress rather than glucose toxicity, mannitol was used as an osmotic control (Figure 7). As a carbohydrate source for endosperm-less seeds, 50 mM glucose was added to mannitol solutions. An application of glucose at 150 mM and/or 200 mM significantly reduced coleoptile growth in all varieties relative to 50 mM glucose. Osmotic controls for 150 mM and 200 mM glucose, which are a mixture of 50 mM glucose and 100 mM or 150 mM mannitol, did not affect coleoptile growth in the four genotypes. These results indicate that growth inhibition observed at high glucose concentrations is not caused by osmotic stress. In summary, the present study has demonstrated that rice varieties without anaerobic germination tolerance are more susceptible to glucose toxicity than those with anaerobic germination tolerance.

## 3. Discussion

This study identified new rice varieties that form long coleoptiles under oxygen deprivation, comparable to well-characterized cultivars with anaerobic germination tolerance, such as Khao Hlan On, Ma-Zhan Red, and Arroz da Terra (Figure 1). These well-studied varieties have been used as donor parents for QTL mapping, identifying the chromosomal regions and genes affecting coleoptile elongation under low oxygen [14,15,17,22]. However, due to the genetic complexity of this trait, identifying novel tolerant accession as additional genetic resources is still imperative, facilitating elucidation of the signaling networks regulating anaerobic germination tolerance in rice.

This study has demonstrated that light and dark cycles increase coleoptile elongation under oxygen deprivation in most rice varieties relative to constant darkness (Figure 2). Under aerobic conditions, constant darkness induces etiolation, characterized by long and thin leaves along with chlorosis, indicating that darkness increases shoot (leaf) elongation. Under anaerobic conditions, however, constant darkness reduced shoot (coleoptile) elongation as compared to light and dark cycles. Although elongated tissues (leaf vs. coleoptile) are distinct under aerobic and anaerobic conditions, growth responses to constant darkness differ depending on oxygen availability. Further studies are required to elucidate the role of long coleoptiles in low-oxygen adaptation in the light and its regulatory mechanisms.

Ethylene and GA are key hormones stimulating shoot elongation under submergence in rice [35]. The present study showed that the application of ACC, an ethylene precursor, and GA alone did not alter coleoptile length of all four varieties under oxygen limitation (Figure 3). The effect of these hormones on coleoptile growth may depend on oxygen concentrations. Indeed, it was shown that ethylene and GA enhanced coleoptile elongation in rice under hypoxia, but not under anoxia [36,37]. It is also anticipated that ethylene performs a positive role in coleoptile elongation when it reaches relatively more aerated layers of floodwaters [6]. Our anaerobic germination system contained approximately 1 ppm of oxygen, which was insufficient for ethylene or GA response. However, the combined application of ACC and GA increased coleoptile elongation even at 1 ppm of oxygen, suggesting that their synergistic effect can augment responsiveness to these hormones under anoxia. Specific processes and members in ethylene and GA signaling pathways can be expected to require oxygen to be functional.

The levels of starch breakdown and soluble carbohydrate accumulation under oxygen deprivation positively correlated with the length of coleoptiles in anaerobically germinated seeds of rice [6,7]. However, in the present study, the degree of starch breakdown in tolerant L202 was similar to or even lower than in intolerant Takanari and IR64 (Figure 4a,c). Moreover, the level of soluble carbohydrate accumulation in tolerant L202 was comparable to or even lower than that in the two intolerant lines (Figure 4b,d). These data indicate that restricted elongation of coleoptiles in intolerant accessions does not result from limited starch hydrolysis under oxygen deprivation. Ethylene and GA increase coleoptile elongation of rice under hypoxia, but not under anoxia [36,37]. Similarly, it is possible that the degree of starch breakdown and soluble carbohydrate accumulation is not a limiting factor for coleoptile elongation under anoxia. In fact, the studies showing the positive correlation between starch breakdown and coleoptile growth were performed under hypoxia [6,7], whereas our experiments were carried out at 1 ppm of oxygen, which is considered anoxia. These observations suggest that regulatory mechanisms underlying coleoptile elongation under hypoxia are not identical to those under anoxia.

As the degree of starch degradation was not associated with anaerobic elongation of coleoptiles in our system, we evaluated the potential impact of soluble carbohydrates highly accumulated within a seedling. To test this, endosperm-less seeds were incubated in deoxygenated water containing a representative soluble sugar, glucose (Figure 6). In all genotypes, low glucose concentrations promoted coleoptile growth, but excessive glucose led to stunted growth, with higher sensitivity to glucose in intolerant than tolerant lines. Osmotic control experiments confirmed that glucose-mediated inhibition of coleoptile growth did not result from osmotic stress (Figure 7). These results suggest that restricted elongation of coleoptiles under oxygen deprivation in intolerant varieties can be attributed to hypersensitivity to soluble sugars accumulated during anaerobic germination.

Previous studies revealed the CIPK15-SnRK1A-MYBS1 cascade, the central mechanism modulating starch degradation and ethanolic fermentation under low oxygen, necessary for anaerobic coleoptile elongation in rice [11,21]. An anaerobic germination regulator, TPP7, is expected to stimulate this cascade through dephosphorylation of T6P, an inhibitor of SnRK1A [15]. Importantly, the activation of the CIPK15-SnRK1A-MYBS1 pathway under oxygen deprivation depends on sugar starvation; supplementation of sucrose deactivates this cascade even under low oxygen [11]. As sugar starvation did not occur in our system, it is most likely that the CIPK15-SnRK1A-MYBS1 cascade is not involved in the regulation of anaerobic coleoptile elongation in the four rice varieties. In the case where soluble sugars are highly accumulated, as observed in this study, sensitivity to soluble sugars, such as glucose, can be a regulatory factor for anaerobic growth of coleoptiles in rice. Further studies on genes controlling soluble sugar sensitivity are required to elucidate molecular mechanisms underlying anaerobic germination tolerance in rice.

## 4. Conclusions

Coleoptile growth under oxygen deprivation is a critical trait influencing anaerobic germination tolerance in rice. Most studies on anaerobic coleoptile growth have been performed under constant darkness because a drilling method was standard practice for direct seeding of rice. However, aerial seeding is gaining popularity, in which seeds are exposed to light on flooded soils during the daytime. Therefore, the present study investigated physiological mechanisms underlying anaerobic coleoptile growth under light and dark cycles. This study has found novel rice accessions, LG and L202, with the ability to develop long coleoptiles under oxygen deprivation, equivalent to well-characterized anaerobic germination tolerant lines. Germination experiments using these two tolerant and two intolerant lines, Takanari and IR64, have demonstrated that light and dark cycles enhance anaerobic coleoptile growth in LG, Takanari, and IR64 compared with constant darkness. Restricted elongation of coleoptiles in intolerant lines was not attributable to limited starch breakdown and soluble sugar accumulation under anaerobic conditions. Instead, stunted coleoptile growth in intolerant lines is likely to result from hypersensitivity to soluble sugars accumulated within anaerobically germinating seeds. Rice varieties with long coleoptiles under oxygen deprivation seem more tolerant to sugar toxicity than those with short coleoptiles, enabling vigorous coleoptile growth at high concentrations of soluble sugars.

## 5. Materials and Methods

### 5.1. Plant Materials and Growth Conditions

This study used 21 varieties of *Oryza sativa* L. Seeds were surface sterilized in 3% (*w*/*v*) sodium hypochlorite and 0.1% (*v*/*v*) Tween-20 for 10 min and rinsed thoroughly with water. Sterilized seeds were immersed in water; only seeds that sank immediately were used for analyses. For anaerobic germination, sterilized seeds were placed in a glass vial (35 mm diameter × 78 mm), which was slowly filled with deoxygenated water (approximately 1 ppm of oxygen) and closed with an air-tight cap. Deoxygenated water was obtained by bubbling nitrogen gas into a 2 L cylinder filled with deionized water for 1 h. Seeds were incubated for up to 5 days at 28 °C under 12 h light (270 µmol/m^2^/s) and 12 h dark cycles or under constant darkness. For endosperm-less seed experiments, dehulled seeds were surface sterilized in the solution mentioned above for 5 min and rinsed thoroughly with water. The endosperm of each seed was removed using a scalpel blade under a dissecting microscope, and endosperm-less seeds were subsequently subjected to anaerobic germination as described above.

### 5.2. Hormone and Carbohydrate Treatments

Hormones and carbohydrates were resolved in submergence water before bubbling nitrogen gas. Dimethyl sulfoxide (DMSO) was used to resolve gibberellin (GA_3_). Mock solutions for GA and ACC + GA treatments contain 0.001% (*v*/*v*) DMSO. ACC, glucose, and mannitol were resolved in deionized water. After deoxygenation, these solutions were used as submergence water for anaerobic germination.

### 5.3. Carbohydrate Assays

Total soluble carbohydrates and starch were quantified using the methods of [38]. Seedling tissue (30 mg) was homogenized in 1 mL of deionized water and incubated at 80 °C for 20 min. Following centrifugation, the supernatant was collected in a new tube. This procedure was repeated twice more, and the three extracts were pooled. The mixed extract was used to quantify total soluble carbohydrates by the anthrone method. The extract (25 µL) was added to 1 mL of 0.14% (*w*/*v*) anthrone solution in 100% sulfuric acid, and the mixture was incubated at 100 °C for 20 min. After cooling, A_620_ of the solution was measured with a spectrophotometer. For starch, the water-insoluble fraction was resuspended in 1 mL of water containing 10 units of heat-resistant α-amylase and incubated at 95 °C for 30 min. After cooling, the suspension was mixed with 25 µL of 1 M sodium citrate (pH 4.8) and 5 units of amyloglucosidase. Following incubation at 55 °C for 1 h, the mixture was centrifuged for 30 min, and the supernatant (10 µL) was used to quantify glucose by the anthrone method. Glucose was used as a standard for total soluble carbohydrate and starch assays.

### 5.4. Statistical Analyses

Statistical analyses were conducted using IBM SPSS Statistics 23 and R version 4.0.3. Student’s *t*-test was performed to compare two datasets. For multiple mean comparisons, ANOVA with Fisher’s least significant difference test or Tukey’s honest significant test was carried out.

## Data Availability

The data are contained within the manuscript and Supplementary Materials.

## Supplementary Materials

The following supporting information can be downloaded at: https://www.mdpi.com/article/10.3390/plants12071565/s1, Figure S1: Percent germination of 18 rice genotypes under non-stress conditions.; Figure S2: Shoot length of 18 rice varieties germinated under anaerobic conditions.

## References

[1] Shiono K., Koshide A., Iwasaki K., Oguri K., Fukao T., Larsen M., Glud R.N. (2022). Imaging the Snorkel Effect during Submerged Germination in Rice: Oxygen Supply via the Coleoptile Triggers Seminal Root Emergence Underwater. Front. Plant Sci..

[2] Kordan H.A. (1976). Anaerobiosis-induced etiolation in light-germinated rice seedlings. Ann. Bot..

[3] Kordan H.A., Ashraf M. (1990). Environmental Anoxia is Unnecessary for Inhibiting Chloroplast Photomorphogenesis in Rice Coleoptiles (*Oryza sativa* L.). J. Exp. Bot..

[4] Perata P., Geshi N., Yamaguchi J., Akazawa I. (1993). Effect of Anoxia on the Induction of Amylase in Cereal Seeds. Planta.

[5] Guglielminetti L., Yamaguchi J., Perata P., Alpi A. (1995). Amylolytic Activities in Cereal Seeds under Aerobic and Anaerobic Conditions. Plant Physiol..

[6] Ismail A.M., Ella E.S., Vergara G.V., Mackill D.J. (2009). Mechanisms Associated with Tolerance to Flooding during Germination and Early Seedling Growth in Rice (*Oryza sativa*). Ann. Bot..

[7] Mondal S., Khan M.I.R., Entila F., Dixit S., Pompe P.C., Panna Ali M., Pittendrigh B., Septiningsih E.M., Ismail A.M. (2020). Responses of AG1 and AG2 QTL Introgression Lines and Seed Pre-Treatment on Growth and Physiological Processes during Anaerobic Germination of Rice under Flooding. Sci. Rep..

[8] Lu C.A., Lim E.K., Yu S.M. (1998). Sugar Response Sequence in the Promoter of a Rice α-Amylase Gene Serves as a Transcriptional Enhancer. J. Biol. Chem..

[9] Lu C.A., Ho T.H.D., Ho S.L., Yu S.M. (2002). Three Novel MYB Proteins with One DNA Binding Repeat Mediate Sugar and Hormone Regulation of α-Amylase Gene Expression. Plant Cell.

[10] Lu C.A., Lin C.C., Lee K.W., Chen J.L., Huang L.F., Ho S.L., Liu H.J., Hsing Y.I., Yu S.M. (2007). The SnRK1A Protein Kinase Plays a Key Role in Sugar Signaling during Germination and Seedling Growth of Rice. Plant Cell.

[11] Lee K.-W., Chen P.-W., Lu C.-A., Chen S., Ho T.-H.D., Yu S.-M. (2009). Coordinated Responses to Oxygen and Sugar Deficiency Allow Rice Seedling to Tolerate Flooding. Sci. Signal..

[12] Lin C.R., Lee K.W., Chen C.Y., Hong Y.F., Chen J.L., Lu C.A., Chen K.T., Ho T.H.D., Yu S.M. (2014). SnRK1A-Interacting Negative Regulators Modulate the Nutrient Starvation Signaling Sensor SnRK1 in Source-Sink Communication in Cereal Seedlings under Abiotic Stress. Plant Cell.

[13] Chen Y.S., Ho T.H.D., Liu L., Lee D.H., Lee C.H., Chen Y.R., Lin S.Y., Lu C.A., Yu S.M. (2019). Sugar Starvation-Regulated MYBS2 and 14-3-3 Protein Interactions Enhance Plant Growth, Stress Tolerance, and Grain Weight in Rice. Proc. Natl. Acad. Sci. USA.

[14] Angaji S.A., Septiningsih E.M., Mackill D.J., Ismail A.M. (2010). QTLs Associated with Tolerance of Flooding during Germination in Rice (*Oryza sativa* L.). Euphytica.

[15] Kretzschmar T., Pelayo M.A.F., Trijatmiko K.R., Gabunada L.F.M., Alam R., Jimenez R., Mendioro M.S., Slamet-Loedin I.H., Sreenivasulu N., Bailey-Serres J. (2015). A Trehalose-6-Phosphate Phosphatase Enhances Anaerobic Germination Tolerance in Rice. Nat. Plants.

[16] Nghi K.N., Tondelli A., Valè G., Tagliani A., Marè C., Perata P., Pucciariello C. (2019). Dissection of Coleoptile Elongation in Japonica Rice under Submergence through Integrated Genome-Wide Association Mapping and Transcriptional Analyses. Plant Cell Environ..

[17] Septiningsih E.M., Ignacio J.C.I., Sendon P.M.D., Sanchez D.L., Ismail A.M., Mackill D.J. (2013). QTL Mapping and Confirmation for Tolerance of Anaerobic Conditions during Germination Derived from the Rice Landrace Ma-Zhan Red. Theor. Appl. Genet..

[18] Mondal S., Khan M.I.R., Dixit S., Cruz P.C.S., Ismail A.M. (2020). Growth, productivity and grain quality of AG1 and AG2 QTLs introgression lines under flooding in direct-seeded rice system. Field Crop. Res..

[19] Castano-Duque L., Ghosal S., Quilloy F.A., Mitchell-Olds T., Dixit S. (2021). An Epigenetic Pathway in Rice Connects Genetic Variation to Anaerobic Germination and Seedling Establishment. Plant Physiol..

[20] Kumar V., Ladha J.K. (2011). Direct seeding of rice: Recent developments and future research needs. Adv. Agron..

[21] Yu S.M., Lee H.T., Lo S.F., Ho T.H.D. (2021). How Does Rice Cope with Too Little Oxygen during Its Early Life?. New Phytol..

[22] Iwata N., Shinada H., Kiuchi H., Sato T., Fujino K. (2010). Mapping of QTLs Controlling Seedling Establishment Using a Direct Seeding Method in Rice. Breed. Sci..

[23] Fukao T., Xu K., Ronald P.C., Bailey-Serres J. (2006). A Variable Cluster of Ethylene Response Factor-like Genes Regulates Metabolic and Developmental Acclimation Responses to Submergence in Rice. Plant Cell.

[24] Fukao T., Bailey-Serres J. (2008). Submergence Tolerance Conferred by Sub1A Is Mediated by SLR1 and SLRL1 Restriction of Gibberellin Responses in Rice. Proc. Natl. Acad. Sci. USA.

[25] Yang C., Lu X., Ma B., Chen S.Y., Zhang J.S. (2015). Ethylene Signaling in Rice and Arabidopsis: Conserved and Diverged Aspects. Mol. Plant.

[26] Sponsel V.M. (2016). Signal achievements in gibberellin research: The second half-century. Annu. Plant Rev..

[27] Hedden P. (2020). The Current Status of Research on Gibberellin Biosynthesis. Plant Cell Physiol..

[28] Watanabe H., Hase S., Saigusa M. (2007). Effects of the Combined Application of Ethephon and Gibberellin on Growth of Rice (*Oryza sativa* L.) Seedlings. Plant Prod. Sci..

[29] Magneschi L., Perata P. (2009). Rice Germination and Seedling Growth in the Absence of Oxygen. Ann. Bot..

[30] Lee K.W., Chen P.W., Yu S.M. (2014). Metabolic Adaptation to Sugar/O2 Deficiency for Anaerobic Germination and Seedling Growth in Rice. Plant Cell Environ..

[31] Perata P., Pozueta-Romero J., Akazawa T., Yamaguchi J. (1992). Effect of Anoxia on Starch Breakdown in Rice and Wheat Seeds. Planta.

[32] Arenas-Huertero F., Arroyo A., Zhou L., Sheen J., León P. (2000). Analysis of *Arabidopsis* glcose insensitive mutants, *gin5* and *gin6,* reveals a central role of the plant hormone ABA in the regulation of plant vegetative development by sugar. Genes Dev..

[33] Moore B., Zhou L., Rolland F., Hall Q., Cheng W.-H., Liu Y.-X., Hwang I., Jones T., Sheen J. (2003). Role of the *Arabidopsis* Glucose Sensor HXK1 in Nutrient, Light, and Hormonal Signaling. Science.

[34] Cho J.I., Ryoo N., Eom J.S., Lee D.W., Kim H.B., Jeong S.W., Lee Y.H., Kwon Y.K., Cho M.H., Bhoo S.H. (2009). Role of the Rice Hexokinases *OsHXK5* and *OsHXK6* as Glucose Sensors. Plant Physiol..

[35] Tamang B.G., Fukao T. (2015). Plant Adaptation to Multiple Stresses during Submergence and Following Desubmergence. Int. J. Mol. Sci..

[36] Horton R.F. (1991). The effect of ethylene and other regulators on coleoptile growth of rice under anoxia. Plant Sci..

[37] Loreti E., Yamaguchi J., Alpi A., Perata P. (2003). Gibberellins Are Not Required for Rice Germination under Anoxia. Plant Soil.

[38] Alpuerto J., Hussain R.M.F., Fukao T. (2016). The key regulator of submergence tolerance, *SUB1A*, promotes photosynthetic and metabolic recovery from submergence damage in rice leaves. Plant Cell Environ..

