# Supplementary material for: The Impact of Carbohydrate Management on Coleoptile Elongation in Anaerobically Germinating Seeds of Rice (Oryza sativa L.) under Light and Dark Cycles
Source: Plants (Basel). 2023 Apr 5;12(7):1565. doi: 10.3390/plants12071565 (PMC10097243; doi:10.3390/plants12071565)
Supplement: Supplementary file 1 [file plants-12-01565-s001.zip › plants-2263301-supplementary.pdf]

plants MDPI

Supplementary Materials

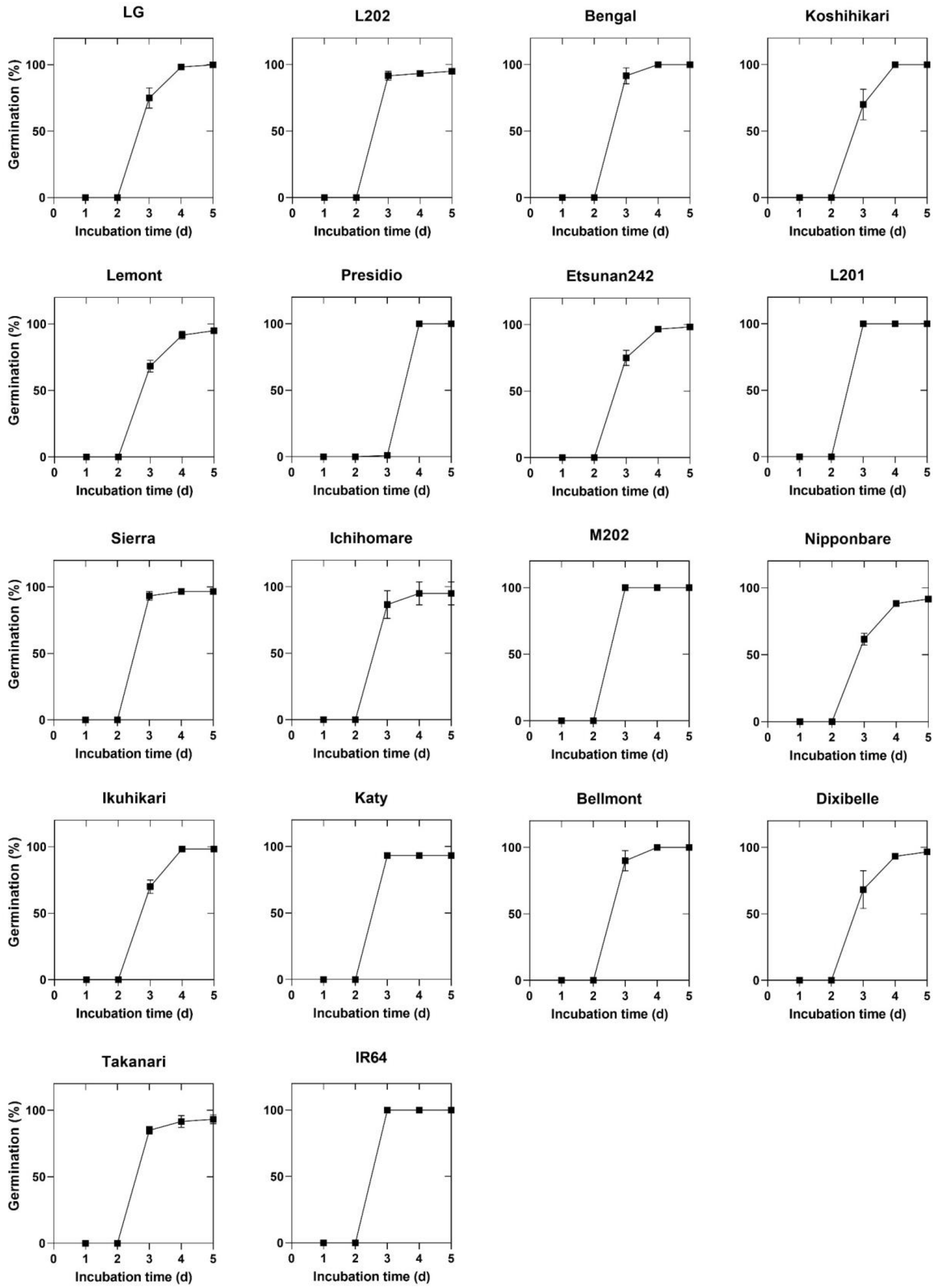

Figure S1. Percent germination of 18 rice genotypes under non-stress conditions. Seeds were incubated at 28 °C (12 h light and 12 h dark) for 5 days. The number of germinated seeds was recorded everyday. Data represent means ± SE (n = 3; 15 seeds per replicate).

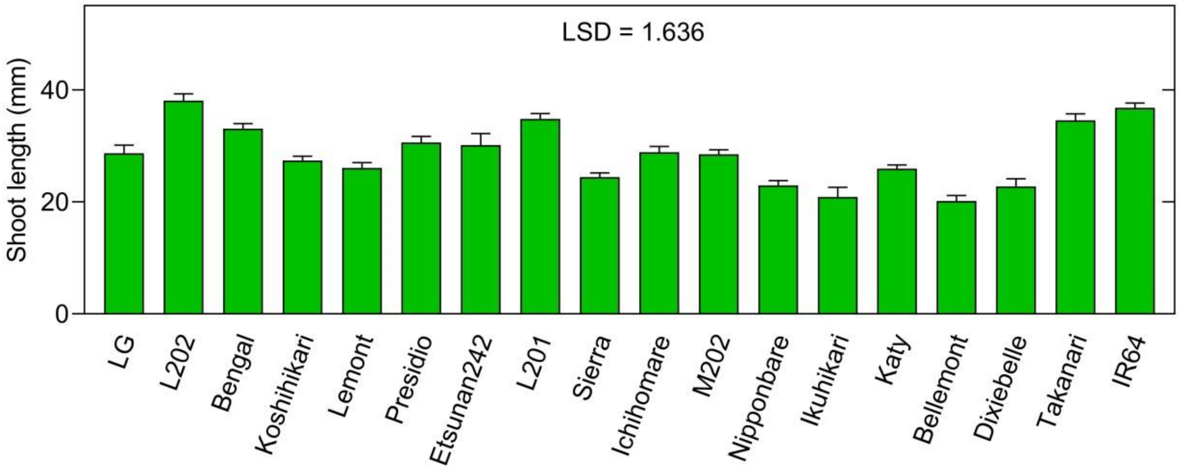

Figure S2. Shoot length of 18 rice varieties germinated under anaerobic conditions. Seeds were incubated at 28 °C (12 h light and 12 h dark) for 5 days. Data represent means ± SE (*n* = 15). Multiple comparisons of means were performed using ANOVA followed by Fisher's least significant difference (LSD) test.
